# Patient dosimetry for total body irradiation using single‐use MOSFET detectors

**DOI:** 10.1120/jacmp.v9i4.2787

**Published:** 2008-11-03

**Authors:** Tina Marie Briere, Ramesh Tailor, Naresh Tolani, Karl Prado, Richard Lane, Shiao Woo, Chul Ha, Michael T. Gillin, A. Sam Beddar

**Affiliations:** ^1^ Department of Radiation Physics The University of Texas M.D. Anderson Cancer Center Houston Texas U.S.A.; ^2^ Division of Radiation Oncology, and Division of Radiation Oncology The University of Texas M.D. Anderson Cancer Center Houston Texas U.S.A.

**Keywords:** total body irradiation, MOSFET, dosimetry

## Abstract

We studied the usefulness of a new type of solid‐state detector, the OneDose single‐use MOSFET (metal oxide semiconductor field effect transistor) dosimeter, for entrance dose measurements for total body irradiation (TBI). The factory calibration factors supplied by the manufacturer are applicable to conventional radiotherapy beam arrangements and therefore may not be expected to be valid for TBI dosimetry because of the large field sizes and extended source‐to‐axis distances used. OneDose detectors were placed under a 1‐cm thick bolus at the head, neck, and umbilicus of 9 patients undergoing TBI procedures. Thermoluminescent dosimeters (TLDs) were placed beside the detectors. We found that the OneDose readings differed from the TLD readings by 4.6% at the head, 1.7% at the neck, and 3.9% at the umbilicus, with corresponding standard deviations of 3.9%, 2.2%, and 2.7%. For all patient measurements, 95% of the OneDose readings fell within 3.3%±6.0% of the TLD readings. Anthropomorphic phantom measurements showed differences of −0.1% at the neck and −1.2% midway between the phantom's carina and umbilicus. Our results suggest that these detectors could be used for TBI quality assurance monitoring, although TLDs should remain the standard when critical dose measurements are performed. If OneDose detectors are to be used for TBI, the use of more than one at each location is strongly recommended. Because the detectors are designed for single use, they cannot be individually calibrated. However, to obtain institution‐specific correction factors for better applicability to TBI dosimetry, measurements of several detectors taken from a particular lot could also be obtained in phantom with the TBI geometry configurations used for patient treatment.

PACS numbers: 87.53.Bn, 85.30.Tv, 87.55.‐x

## I. INTRODUCTION

Over the past few years, interest in MOSFET (metal oxide semiconductor field effect transistor) dosimetry for radiotherapy beams has been increasing, stimulated by new technology developments leading to improved types of detectors.^(^ ^1^ ^–^ ^11^ ^)^ One of these is the OneDose detector (manufactured by Sicel Technologies, Morrisville, NC; distributed by MedTec, Orange City, IA), which has been a subject of interest for standard dosimetry,^(7)^ quality assurance of megavoltage photon beams,^(11)^ and patient dosimetry for total body irradiation (TBI) procedures.^(6)^ For conventional geometries under full buildup conditions, the manufacturer states that the accuracy^(12)^ for these detectors is

- ±1 cGy at doses under 20 cGy, and
- ±5% at doses of 20 – 500 cGy (2 σ).

Two important characteristics of these detectors are that they are wireless, and they can be read immediately after completion of a treatment procedure. The latter characteristic would be particularly advantageous for short treatment courses, including entrance dose measurements for TBI procedures and stereotactic treatments.

Dosimetry for TBI is generally performed using silicon diodes^(^ ^13^ ^–^ ^15^ ^)^ or thermoluminescent dosimeters (TLDs).^(14)^ ^,^ ^(16)^ At our institution, TLDs in powder form are the standard detector used for patient dosimetry. The goal of the present work was to determine if the OneDose detector can be used clinically as an alternative to TLDs for monitoring and measuring TBI doses that are delivered at various anatomic sites.

## II. METHODS AND MATERIALS

The OneDose detector system has been described in detail elsewhere.^(7)^ In our study, the OneDose detectors, which were provided by the manufacturer and its distributor, were selected from four separate lots. Each lot of detectors was calibrated by the manufacturer with Co60 under full buildup conditions.^(12)^ The detectors were zeroed before irradiation. The default calibration factor was used for all measurements (that is, no corrections were applied for beam energy, field size, or distance). Because LiF thermoluminescent dosimeters (TLDs) have been the main dosimeters used for patient dosimetry at our institution as well as at other centers, they were used as the standard detectors for comparison. The LiF:Mg,Ti powder (TLD‐100: Thermo Scientific, Waltham, MA) was placed in flat packs with approximate dimensions of 0.7×0.7 cm. The typical weight of the powder in each flat pack was 0.023 g. At the time of purchase, the batch of TLD powder was calibrated under Co60 at known doses to obtain the appropriate linearity corrections. The OneDose detector and two TLDs were attached to a 3×3−cm piece of Superflab (Fluke Biomedical, Everett, WA) bolus with a thickness of 1 cm. To measure entrance dose at several sites on the patient's surface, the bolus was taped to the patient's anterior skin surface at the forehead, neck, and umbilicus so that the front surface of the OneDose detector directly faced the beam.

**Figure 1:**
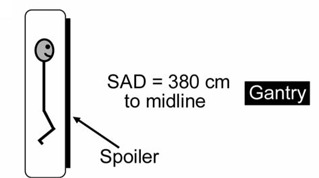
The most common total body irradiation configuration used at our institution: a couch unit in which the patient lies on his or her side at a source‐to‐axis distance (SAD) of 380 cm. An 18‐MV beam is used, and the collimator is rotated by 45 degrees and set to 40×40 cm.

Study patients (N=9) were placed in the TBI configuration as illustrated in Fig. 1. This consists of a couch unit on which the patient lies on his or her side at a source‐to‐axis distance of 380 cm. A rice bag was placed on the patient's neck to improve dose homogeneity in the couch setup. All patients were treated with 18‐MV photons, and the collimator was rotated by 45 degrees and set to 40×40 cm. The actual field size at the patient's extended source‐to‐axis distance was more than 150×150 cm. Note that the beam energy is different from that described in an earlier study of the OneDose detector applied to TBI.^(6)^ Although all patients were treated with both anterior‐posterior and posterior‐anterior fields, the detectors were removed immediately following irradiation of the anterior‐posterior field. Table 1 shows data relevant to the procedure.

**Table 1:** Treatment data

Beam energy	18 MV
Measured fields	Anterior–posterior
Source‐to‐axis distance	380 cm
Prescribed dose per field	75 cGy (n=1)
at patient midsection	87.5 cGy (n=3)
	150 cGy (n=5)
Monitor units	1140–2310
Patient thickness	14.5–28 cm (average: 22 cm)
Patient weight	21–128 kg (average: 72 kg)

The OneDose detectors were read within 5 minutes of completion of the irradiation.

Following completion of the TBI procedure, three TLDs to be used as the calibration standards were irradiated under the same 18‐MV beam, but with a source‐to‐surface distance (SSD) of 100 cm, a depth of 3.3 cm, and a field size of 10×10 cm. The TLDs were read at least 3 days after irradiation.

The average sensitivity from all the standards was used to calculate the TLD dose. This approach assumes that the sensitivity of the TLD powder is constant and averages the effect of daily fluctuations in machine output. A single OneDose detector was irradiated with the standards to compare the dose readings obtained by both detectors (for determining consistency) and to verify that the OneDose detectors used in this study from the different lots were all within the manufacturer's specifications.

Finally, phantom measurements were performed using a three‐dimensional anthropomorphic torso phantom (Model 602: Computer Imaging Reference Systems, Norfolk, VA). The phantom had a thickness of 21 cm, similar to the 9‐patient average. The monitor units were based on a prescribed dose of 150 cGy. Four OneDose detectors and four TLDs were placed at the phantom's midline between the carina and umbilicus, and four more detectors of each kind were placed at the phantom's neck and then covered with a rice bag. The OneDose detectors were selected from a fourth lot that was not used for any patient measurements. As with the patient measurements, all detectors were covered with 1‐cm bolus. The phantom was placed on its side and irradiated in the patient geometry described earlier.

## III. RESULTS AND DISCUSSION

For the phantom measurements, the standard deviation in the sensitivity of the TLDs used as the calibration standards was 1.8%. The results for the OneDose detectors placed on the abdominal phantom in the TBI geometry showed an average difference from the TLD readings of −1.2% at the neck and −0.1% at the midline. For both sites, the standard deviation for the OneDose readings was 0.4%. A check of the detectors irradiated at standard geometry showed an average difference of −1.1% and a standard deviation of 1.6%.

**Figure 2:**
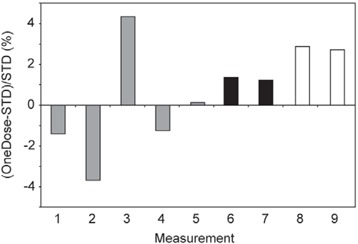
Percent deviation of the OneDose detectors from the thermoluminescent dosimeter standard (STD) for the 9 calibration measurements taken at conventional geometry. OneDose detectors taken from lot 1 are shown in gray, those from lot 2 in black, and those from lot 3 in white.

For the patient measurements, the standard deviation in the sensitivity of all the TLDs used as the calibration standards was 1.9%. The results for the OneDose detectors irradiated at conventional geometry show agreement with the TLD readings with an average difference of 0.7% and a maximum difference of 4.3%, well within manufacturer's specifications (Fig. 2). The standard deviation in the differences of these measurements was 2.5%. These results are in line with earlier results reported for conventional geometries.^(7)^ ^,^ ^(11)^

**Figure 3:**
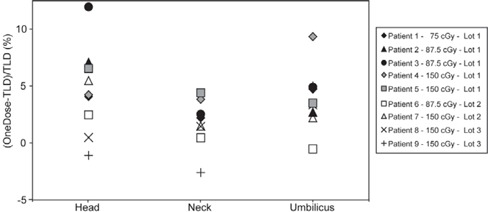
Percent deviation from thermoluminescent dosimeter (TLD) measurements for the 9 total body irradiation patients. The detectors were placed at the head, neck, and umbilicus of each patient. The prescribed dose to the patient's midsection was 75 cGy for patient 1, 87.5 cGy for patients 2 – 4, and 150 cGy for patients 5 – 9. Patients 1 – 5 (filled symbols) received detectors from lot 1, patients 6 and 7 from lot 2 (open symbols), and patients 8 and 9 (crosses) received detectors from lot 3.

Fig. 3 shows the results for all the TBI patient measurements. For the patient with a prescribed dose of 75 cGy at the midsection, the measured entrance doses ranged from 92 cGy to 102 cGy; for the patients with prescribed doses of 87.5 cGy, the measured doses ranged from 100 cGy to 118 cGy; and for a prescribed dose of 150 cGy, the measured doses ranged from 164 cGy to 215 cGy. One outlier was considered spurious and was therefore excluded. The average difference of the OneDose detectors from the TLD readings was 4.6% at the head, 1.7% at the neck, and 3.9% at the umbilicus, with corresponding standard deviations of 3.9%, 2.2%, and 2.7%. The average difference between the two TLD readings at each site was 1.1%.

Comparing the results with respect to prescribed dose (Fig. 3), no clear relationship was observed between measured and delivered dose as the prescribed dose increased. The differences between the OneDose and TLD doses measured at the head and umbilicus are higher than those measured at the neck, and this trend is similar to the results from the phantom measurements discussed earlier, although the differences were greater in the patient measurements. It is possible that the differences in the results for the neck and for the head and umbilicus could be a result of the additional buildup from the rice bag placed on the patient's neck. Because of the use of a 1‐cm acrylic spoiler plate, the bolus, and the large field size, the detectors placed on the head and umbilicus should have been in a relatively flat dose region close to $d_{\max}$. Thus, although the OneDose detectors have an inherent buildup of 1.2 mm,^(7)^ we predict that the dose absorbed by the OneDose detectors and TLDs should have been nearly the same at the head and umbilicus. The addition of the rice bag (with an approximate thickness of 2 cm) put those detectors beyond $d_{\max}$, where the additional inherent buildup of the OneDose detectors should have led to a negligible decrease in the percent difference (0.2%/mm) as compared with the measurements close to $d_{\max}$. However, the difference between the neck and the head and umbilicus is larger than expected, and we therefore suspect the difference to be the result of statistical uncertainty of both the TLDs and the OneDose detectors.

Our results show a greater deviation than that seen in an earlier study that used a 6‐MV beam for TBI.^(6)^ For the 2 patients in that study, with detectors (all from the same lot) placed at either 7 or 11 sites, the authors found the detectors to agree with TLD measurements within 3.9%. Studying a greater number of patients over a period of months, we found that more than 20% of measurements were outside the range of ±5% relative to the TLDs. Thus, although the previous study suggests that the OneDose detector provides an accuracy comparable to that of TLDs, the present results suggest that the OneDose may not be as reliable as TLDs for TBI measurements and that TLDs should remain the standard when critical dose measurements are performed. However, the ease of use and convenience of the OneDose detectors makes them very useful for most TBI measurements.

We also observed some dependence on the lot from which the OneDose detectors were selected. For instance, detectors selected from lot 1 tended to read higher than those from lots 2 and 3 (Fig. 3). However, we should note that the manufacturer's recommended energy correction factor for conventional 18‐MV X‐ray beams was nearly the same for lots 1 and 2 (1.001 and 0.999 respectively), but significantly larger for lot 3 (1.026). The results are converse to those obtained at standard geometries (Fig. 2), where 4 of the 5 detectors from lot 1 showed an under‐response. For OneDose lot 4, used for the anthropomorphic phantom measurements, the manufacturer's correction factor was 1.016, and the detectors showed a slight under‐response of −1.2% for the midline measurements, demonstrating further lot dependence. As mentioned earlier, these suggested correction factors were not used in this study because they do not apply to TBI beam configurations, including energy, dose rate, field size, and source‐to‐surface distance.

Examining Fig. 3, it could be seen that a global correction of about 3.3% would bring the calibration of the OneDose detector to a level sufficient for patient dosimetry during TBI procedures. Using this correction factor, 95% of the data points would lie within ±6.0% of the TLD readings. However, this correction factor would depend on the setup used at each particular institution. For normal clinical use, we would recommend that these detectors be calibrated in phantom using the TBI geometry configurations used for patient treatment to obtain institution‐specific correction factors for TBI dosimetry. Considering our results for the phantom measurements and the variation observed between manufacturing lots for the patient measurements, these correction factors should ideally be validated for each individual manufacturing lot. To improve the accuracy of the readings and to indicate if a particular detector reading is an unexpected outlier, we recommend the use of two detectors at each site.

## IV. CONCLUSIONS

The OneDose detector system was clinically evaluated for TBI patient dosimetry in 9 patients. Results of measurements, without the application of any correction factor, showed a difference of between 1.7% and 4.6% on average in the OneDose and TLD readings. For all patient measurements, 95% of the OneDose readings fell within 3.3%±6.0% of the TLD readings. The main advantage of the OneDose detectors over TLDs is that they can be read immediately after irradiation, and this advantage may be particularly important for short treatment courses such as twice‐daily TBI regimens. However, these detectors will not supplant the other commonly used detectors that have been proven to be successful for TBI measurements.

## ACKNOWLEDGMENTS

The authors are grateful to Sicel Technologies and MedTec for supplying the reader and detectors used in this study.

## CONFLICT OF INTEREST STATEMENT

In the past, the first and senior authors had a sponsored research agreement with Sicel Technologies to study their implantable sensors.

## References

[1] Rowbottom CG , Jaffray DA . Characteristics and performance of a micro‐MOSFET: an “imageable” dosimeter for image‐guided radiotherapy. Med Phys. 2004;31(3):609–615. 15070261 10.1118/1.1649532

[2] Jornet N , Carrasco P , Jurado D , Ruiz A , Eudaldo T , Ribas M . Comparison study of MOSFET detectors and diodes for entrance *in vivo* dosimetry in 18 MV X‐ray beams. Med Phys. 2004;31(9):2534–2542. 15487735 10.1118/1.1785452

[3] Scarantino CW , Ruslander DM , Rini CJ , Mann GG , Nagle HT , Black RD . An implantable radiation dosimeter for use in external beam radiation therapy. Med Phys. 2004;31(9):2658–2671. 15487749 10.1118/1.1778809

[4] Cygler JE , Saoudi A , Perry G , Hallil A , Brown M , Thomson I . Measurement of urethral dose profiles in prostate brachytherapy using a linear MOSFET array dosimeter. Radiother Oncol. 2004;71(Suppl 2):592–593.

[5] Beddar AS , Salehpour M , Briere TM , Hamidian H , Gillin MT . Preliminary evaluation of implantable MOSFET radiation dosimeters. Phys Med Biol. 2005;50(1):141–149. 15715428 10.1088/0031-9155/50/1/011

[6] Best S , Ralston A , Suchowerska N . Clinical application of the OneDose Patient Dosimetry System for total body irradiation. Phys Med Biol. 2005;50:5909–5919. 16333163 10.1088/0031-9155/50/24/010

[7] Halvorsen PH . Dosimetric evaluation of a new design MOSFET in vivo dosimeter. Med Phys. 2005;32(1):110–117. 15719961 10.1118/1.1827771

[8] Scalchi P , Francescon P , Rajaguru P . Characterization of a new MOSFET detector configuration for in vivo skin dosimetry. Med Phys. 2005;32(6):1571–1578. 16013716 10.1118/1.1924328

[9] Briere TM , Beddar AS , Gillin MT . Evaluation of pre‐calibrated implantable MOSFET radiation dosimeters for megavoltage photon beams. Med Phys. 2005;32(11):3346–3349. 16370421 10.1118/1.2065447

[10] Scarantino CW , Rini CJ , Aquino M , et al. Initial clinical results of an in vivo dosimeter during external beam radiation therapy. Int J Radiat Oncol Biol Phys. 2005;62(2):606–613. 15890606 10.1016/j.ijrobp.2004.09.041

[11] Briere TM , Lii J , Prado K , Gillin MT , Beddar AS . Single‐use MOSFET radiation dosimeters for the quality assurance of megavoltage photon beams. Phys Med Biol. 2006;51:1139–1144. 16481683 10.1088/0031-9155/51/5/006

[12] Sicel Technologies . OneDose dosimeter quick reference guide. Morrisville (NC): Sicel Technologies.

[13] Mangili P , Fiorino C , Rosso A , et al. In‐vivo dosimetry by diode semiconductors in combination with portal films during TBI: reporting a 5‐year clinical experience. Radiother Oncol. 1999;52:269–276. 10580875 10.1016/s0167-8140(99)00104-8

[14] Essers M , Mijnheer BJ . In vivo dosimetry during external photon beam radiotherapy. Int J Radiat Oncol Biol Phys. 1999;43(2):245–259. 10030247 10.1016/s0360-3016(98)00341-1

[15] American Association of Physicists in Medicine (AAPM), Radiation Therapy Committee , Task Group 62. Diode in vivo dosimetry for patients receiving external beam radiation therapy. AAPM report No. 87. Madison (WI): Medical Physics Publishing; 2005.

[16] American Association of Physicists in Medicine (AAPM) , Radiation Therapy Committee, Task Group 29. The physical aspects of total and half body photon irradiation. AAPM report No. 17. New York (NY): American Institute of Physics; 1986.

